# Two new *Megalothorax* species of the *minimus* group (Collembola, Neelidae)

**DOI:** 10.3897/zookeys.554.6069

**Published:** 2016-01-18

**Authors:** Clément Schneider, David Porco, Louis Deharveng

**Affiliations:** 1 Institut de Systématique, Evolution, Biodiversité, ISYEB - UMR 7205 - CNRS, MNHN, UPMC, EPHE, Museum national d’Histoire naturelle, Sorbonne Universités, 45 rue Buffon, CP50, F-75005 Paris, France; 2 Université de Rouen, Laboratoire ECODIV, Bâtiment IRESE A, Place Emile Blondel, 76821 Mont Saint Aignan Cedex, France

**Keywords:** Springtails, Neelipleona, description, chaetotaxy, taxonomy, DNA barcodes, labrum

## Abstract

Two new *Megalothorax* species, *Megalothorax potapovi* **sp. n.** from the Russian Far East and *Megalothorax sanguineus* **sp. n.** from the French Pyrénées are described. The two new species have a set of morphological characters (including a smooth mucro) that places them among the *minimus* group *sensu* Schneider and D’Haese (2013). *Megalothorax potapovi* characteristics include dorsal protuberance on forehead, peculiar chaetotaxy of antenna III and strong lanceolate chaetae on body. *Megalothorax sanguineus* characteristics include strong red pigmentation, large network of integumentary channels on head and elongated apex of the two postero-distal spines of dens. The DNA barcodes (cytochrome oxidase subunit I–COI) of the two species are also provided and analyzed among a broader sampling of the genus in order to support further their specific status. A special focus is given to the labral morphological characteristics. Pseudopores-like elements are reported for the first time in the genus. Positions of the τ-chaetae near the dorsal sensory field of thorax II are compared for several species of the genus.

cytochrome oxidase subunit I–

## Introduction

During an expedition organized by Mikhail Potapov in Primorye (Russian Far East) in 2004, a large number of samples yielded a huge diversity of Collembola. Several of the most remarkable taxa collected have been recently described, like the new genus *Sensillonychiurus* with three new species (Pomorski and Sveenkova 2006), a genus that was subsequently retrieved in Northeastern China (Sun et al. 2013) and in other regions of northern Russia (Babenko et al. 2011). The Sino-Korean subfamily Caputanurininae was also reported for the first time for Russia (Deharveng et al. 2010) with two new species. Here, we report a morphologically remarkable new species of the genus *Megalothorax* Willem, 1900 (Neelidae, Neelipleona): *Megalothorax potapovi* sp. n. On the other end of the Palaearctis, the faunistical survey of the Pyrenean peatland of Bernadouze yielded another new species of *Megalothorax*, *Megalothorax sanguineus* sp. n. The two new species are described in the present paper, along with their barcode sequences (COI–mitochondrial cytochrome oxidase subunit I). Their genetic divergence levels with other *Megalothorax*
molecular operational taxonomic units (MOTUs) are assessed and discussed. A special focus is given to the labral morphological characteristics. Pseudopores-like elements are reported for the first time in the genus. Positions of the τ-chaetae near the dorsal sensory field of Th. II are compared for several species of the genus.

## Material and methods

### Sampling

*Megalothorax potapovi* sp. n. The specimens were obtained from a 2000 cm^3^ sample of forest litter from Primorye, that were processed for fauna extraction in a field laboratory of Anisimovka. The litter sample was dried for 8 days on a Berlese funnel without heating.

*Megalothorax sanguineus* sp. n. The two sampled sites are located at medium elevation in Ariège Pyrenees (France): in very humid mossy habitats near the peat-bog of Bernadouze, and from humid litter at Osque du Couret. Samples were processed in the lab on Berlese funnels in the same conditions as above.

### Morphology

Specimens were preserved in 95% ethanol then cleared in lactic acid and finally mounted on microscope slides in Marc André II medium. They were examined using a Leica DMLB compound microscope with differential phase contrast optics at magnifications ranging from 250 to 1000. Drawings were made with a drawing tube and vectorized with Inkscape. For Scanning Electronic Microscope (SEM) observations, specimens were dehydrated in 100% ethanol, before critical point drying (Emitech K850) and gold coating (Jeol JFC-1200) and observations were performed with a SEM Jeol 840A. *Megalothorax sanguineus* sp. n. was not observed with SEM, however we were able to locate all the positions of the τ-chaetae (trichobothria) with the optical microscope.

### DNA barcode

The standard DNA barcode (658bp of the COI–mitochondrial cytochrome oxidase subunit I gene, Hebert et al. 2003) was sequenced for the 2 new species (Table 4).

DNA was extracted from entire specimens in 30μl of lysis buffer (http://www.ccdb.ca/docs/CCDB_DNA_Extraction.pdf) and proteinase K incubated at 56 °C overnight. DNA extraction followed a standard automated protocol using 96-well glass fibre plates (Ivanova et al. 2006). Specimens were recovered after DNA extraction using a specially designed work flow allowing their morphological examination (Porco et al. 2010). The 5’ region of COI used as a standard DNA barcode was amplified using M13 tailed primers LCO1490 and HCO2198 (Folmer et al. 1994). Samples that failed to generate an amplicon were subsequently amplified with a pair of internal primers combined with full length ones (C_LepFolF/C_LepFolR) (Ivanova - published on http://www.boldsystems.org). The standard PCR reaction protocol of the Canadian Center for DNA Barcoding was used for amplifications (http://www.dnabarcodes2011.org/conference/preconference/CCDB-Amplification-animals.pdf), and products were checked on a 2% E-gel 96Agarose (Invitrogen). Unpurified PCR amplicons were sequenced in both directions using M13 tailed primers, with products subsequently purified using Agencourt CleanSEQ protocol and processed using BigDye version 3.1 on an ABI 3730 DNA Analyzer (Applied Biosystems). Sequences were assembled and edited with Sequencher 4.5 (GeneCode Corporation, Ann Arbor, MI, USA). The alignment was obtained using BIOEDIT version 7.0.5.3 (Hall 1999). Sequences are publicly available on GenBank (JN298074-JN298078, JN970909-JN970929, KC900191-KC900205, KR736063-KR736070) and on BOLD at the following doi: 10.5883/DS-MEGAMIN (Table 4).

### Data analyses

Forty-nine specimens of *Megalothorax* dataset representing 14 morphologically recognized species were selected, 36 from Schneider et al. 2011, Schneider and D’Haese 2013 and 13 specimens belonging to the two new species (Table 4).

Distance analyses were performed with MEGA6 (Tamura et al. 2013), utilizing a Neighbor-Joining (Saitou and Nei 1987) algorithm with the Kimura-2 parameter model (Kimura 1980) to estimate genetic distances. The robustness of nodes was evaluated through bootstrap re-analysis of 1000 pseudoreplicates. Molecular Operational Taxonomic Units (MOTUs) were defined with the software ‘mothur’ (Schloss et al. 2009).

### Terminology

A nomenclature for the integumentary crests on the labrum is introduced (Fig. 13A–F). Crests are defined as integumentary processes with an apical line of primary grains. The longitudinal crests separating the m-row of chaetae are named *ml1*–*3*, when present the transversal crests in posterior position to a chaetae of the m-row are named *mt* and numbered after the chaetae position (*mt2* posterior to chaeta *m2*), the antero-median transversal crest separating the m-row from the a-row is named *amt*, and can be further separated in *amt0*–*2* numbered after the chaetae of m-row position. The longitudinal anterior crests separating the a-row are named *al1*–*3*. The transversal crest anterior to the a-row is named *at*—theoretically with the subdivisions *at0*, *at1*, *at2* though we could only observe the *at2* region in *Megalothorax minimus* Willem, 1900.

Head chaetotaxy (Fig. 14) and antenna chaetotaxy (Fig. 15) follow Schneider (in press), trunk chaetotaxy (Fig. 16) follows Schneider and D’Haese (2013). The four swollen chaetae of Ant. III sensory organ are named *S1*–*S4* after Deharveng (1978). We avoid to use the term ‘sensilla’ to designate some chaetae with peculiar shape and light refraction (e.g. Massoud and Ellis 1977), and use instead the following categories defined by Schneider (in press): (i) s-chaetae for the short swollen chaetae of the trunk; (ii) τ-chaetae for the long and thin chaetae of the trunk (shaped as trichobothria in *Megalothorax*); (iii) S-chaetae for the swollen chaetae of the antenna; (iv) neosminthuroid chaetae for the special chaetae of Abd. IV sternum as defined in Richards (1968), Betsch (1980) and Schneider and D’Haese (2013). Ordinary chaetae are simply referred as chaetae. Nomenclature of the claw follows Schneider (in press), based on Denis (1948) and Schneider and D’Haese (2013). The presence or absence of specific chaetae is described in reference of the chaetotaxic pattern of *Megalothorax minimus*. Reference to *Megalothorax minimus* in this work is always sensu Schneider and D’Haese (2013).

### Abbreviations and symbols in text and figures

**Crests on the anterior process of the labrum**: *al2*, *3* = anterior longitudinal, *ml1*–*3* = posterior longitudinal, *amt0*–*2* = antero-median transversal, *mt2* = posterior transversal. **Antenna**: Ant. I–IV = antennomere I to IV, *S1*–*S4* = S-chaetae of Ant. III; or = Ant. IV organite; *S*, *Sx*, *Sy* = S-chaetae of Ant. IV. **Trunk**: Th. I–III = thoracic segment I to III; Abd. I–VI = abdominal segment I to VI; *av* = chaetae of anal valve; *s1*, *s2* = s-chaetae; *sm* = special chaetae of male Abd. VI sternum; τ = τ-chaetae; *wrc1*–*wrc8* = free wax rod secretory element 1 to 8. **Claw**: *la*, *lp*, *Ba*, *Bp* = auxiliary lamellae and crest of unguis; *Ca*, *Cp* = anterior and posterior crests of unguiculal lamella. **Misc**: dp = dens proximal, dd = dens distal, *sf1*–*6* = sensory field 1 to 6.

## Taxonomy

### Megalothorax potapovi sp. n.

Taxon classificationAnimaliaCollembolaNeelidae

http://zoobank.org/4E6BA360-2EDF-49F3-AF77-54B250A9C490

Figs 1 , 2 , 3 , 4 , 5 , 6 , 7 , 13A–C , 14A, B , 15 , 16A

#### Material examined.

Holotype: male on slide (MNHN-EA040223), Russia: Primorye: south of Posyet: peninsula facing to the town; 130.8034°E, 42.5709°N; alt=30 m; 28.ix.2004; Berlese extraction, forest litter; Louis Deharveng and Anne Bedos leg (RU-120) [MNHN]. Paratypes: 2 males and 5 females on slides (MNHN-EA040224–229), same data as the holotype [MNHN]; 1 specimen (sex unknown) on mount for SEM (MNHN-EA041012), same data as the holotype [MNHN].

#### Diagnosis.

Whitish in alcohol. Presence of median integumentary protuberance in front of chaeta *a0* on forehead. Presence of chaeta *X* on Ant. IV. Labium: basomedian fields with 3 + 3 chaetae, basolateral fields with 1 + 1 chaetae. Integumentary channels as a paired tree on posterior part of the head, absent on anterior part, connection of channels with *linea ventralis* circular. Some chaetae enlarged and lanceolate, of which 5 + 5 dorsal, posterior on head and 2 + 2 on Th. II tergum. Inner chaeta of sensory field 2 slender with blunt apex, all inner chaetae of sensory fields 3–6 short flam-shaped. Dorsal abdominal s-chaetae *s2* globular, absence of dorsal abdominal s-chaetae *s3*. Abd. I to V terga with 18 + 18 ordinary chaetae. Each claw of ordinary morphology, subequal. Tenaculum with 3 + 3 teeth. Abd. IV sternum with 2 + 2 chaetae. Mucro lamellae smooth, thin.

#### Description.

**Figure 1.**
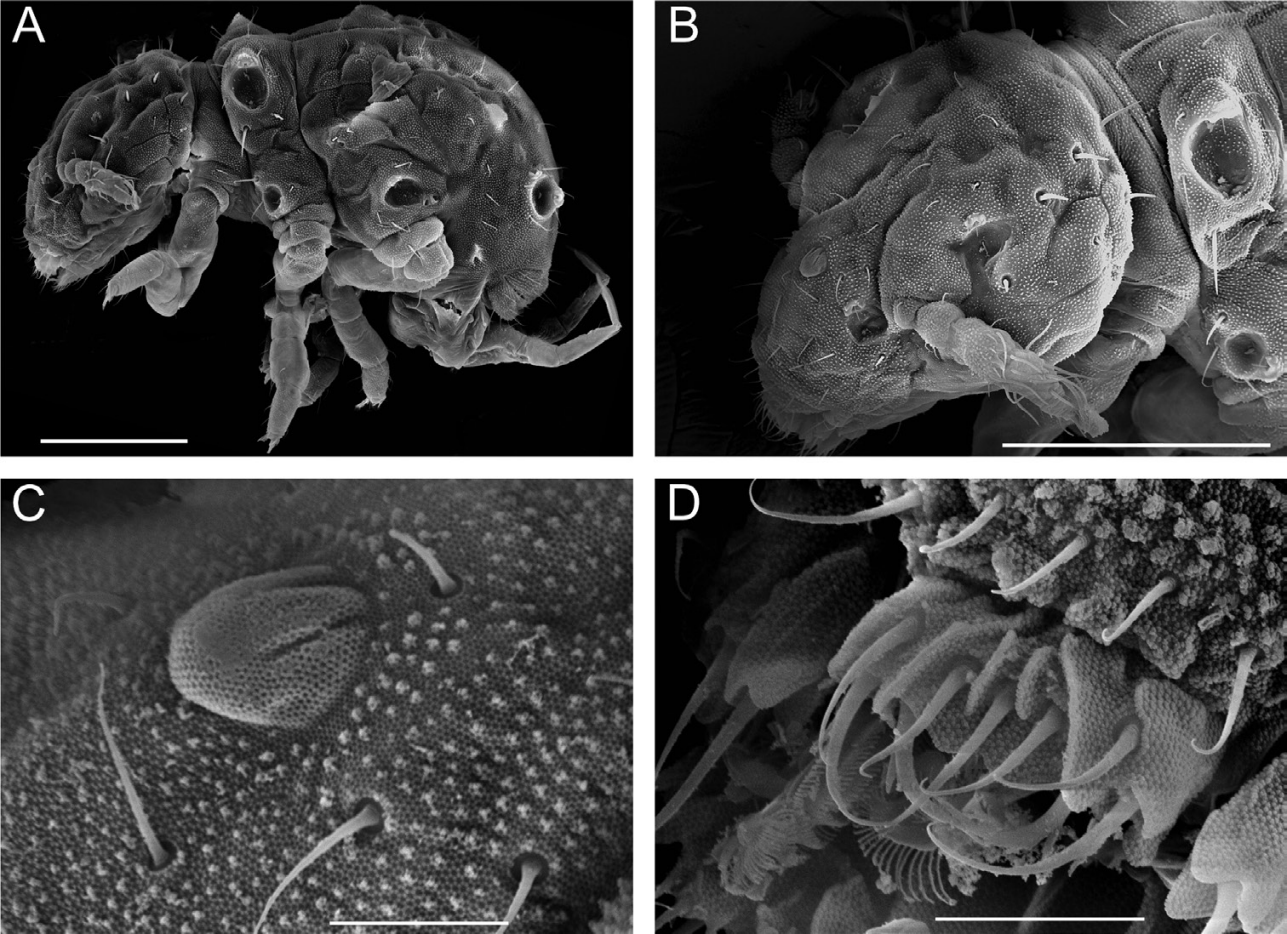
*Megalothorax potapovi* sp. n. **A** Whole body, lateral view **B** head, dorso-lateral view **C** integumentary protuberance on forehead, dorso-lateral view **D** labrum, dorsal view. Scale bars: 100 μm (**A, B**); 10 μm (**C, D**).

**General aspect.** Habitus and segmentation typical of the genus (Fig. 1A). Length from labrum to anus: ~380 μm. Specimens whitish in alcohol. Body chaetotaxy sparse including chaetae, s-chaetae, τ-chaetae as trichobothria, neosminthuroid chaetae, wax rod secretory elements and special swollen chaetae within *sf2*–*6*. Length of chaetae ranging from microchaetae [5–9 μm] to mesochaetae [10–14 μm] and macrochaetae [15–24 μm]. Shape of chaetae ranging from simple to lanceolate. Greatest chaetae being macrochaetae *a4* and *a7* on Th. II tergum (24 μm; Fig. 5F, G).

**Figure 2.**
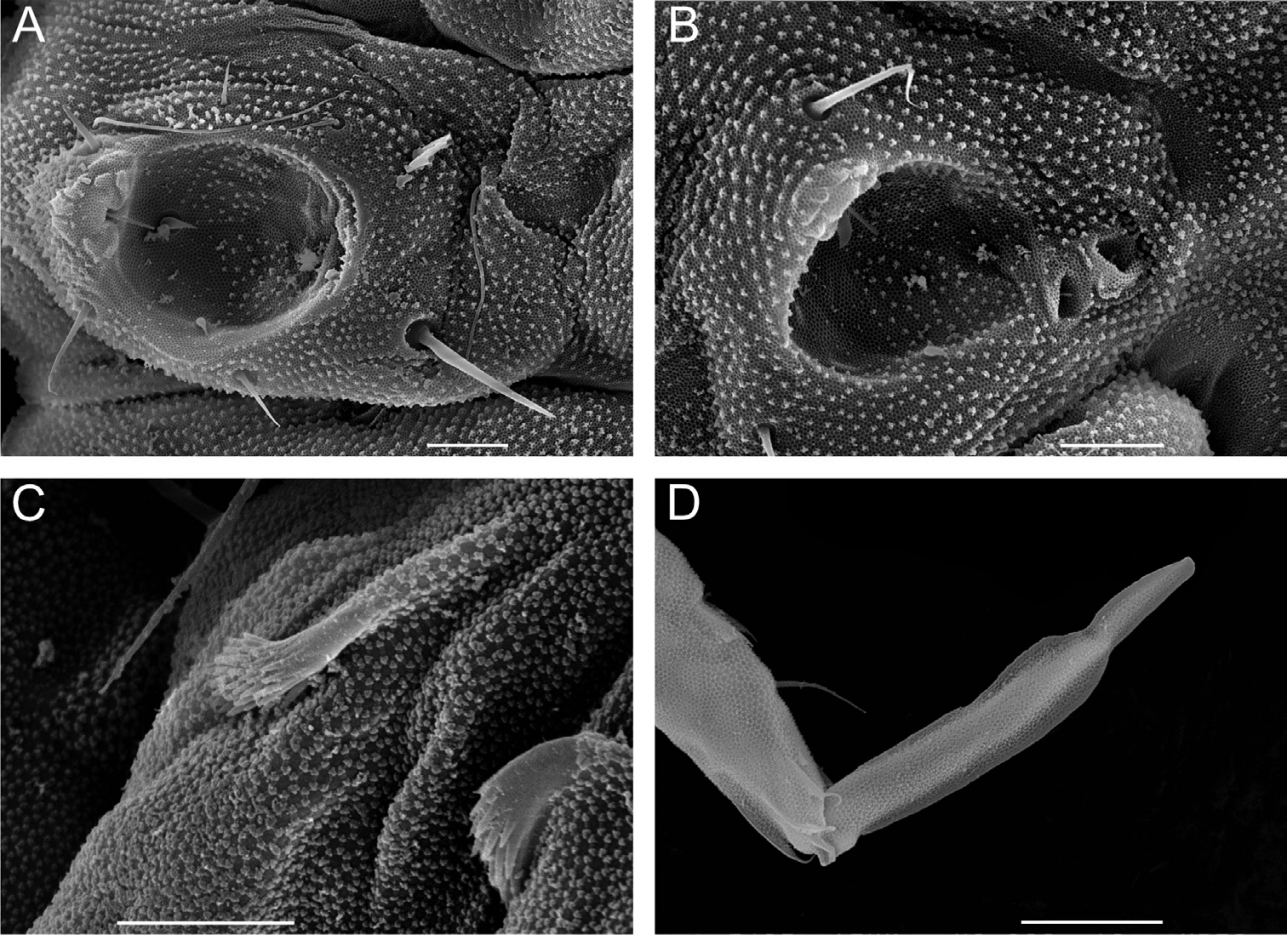
*Megalothorax potapovi* sp. n. **A** *sf3* on Th. II tergum, dorsal view **B** *sf5* on Th. III tergum, lateral view **C** neosminthuroid chaetae on Abd. IV sternum, lateral view **D** mucro, lateral view. Scale bars: 10 μm (**A–D**).

**Figure 3.**
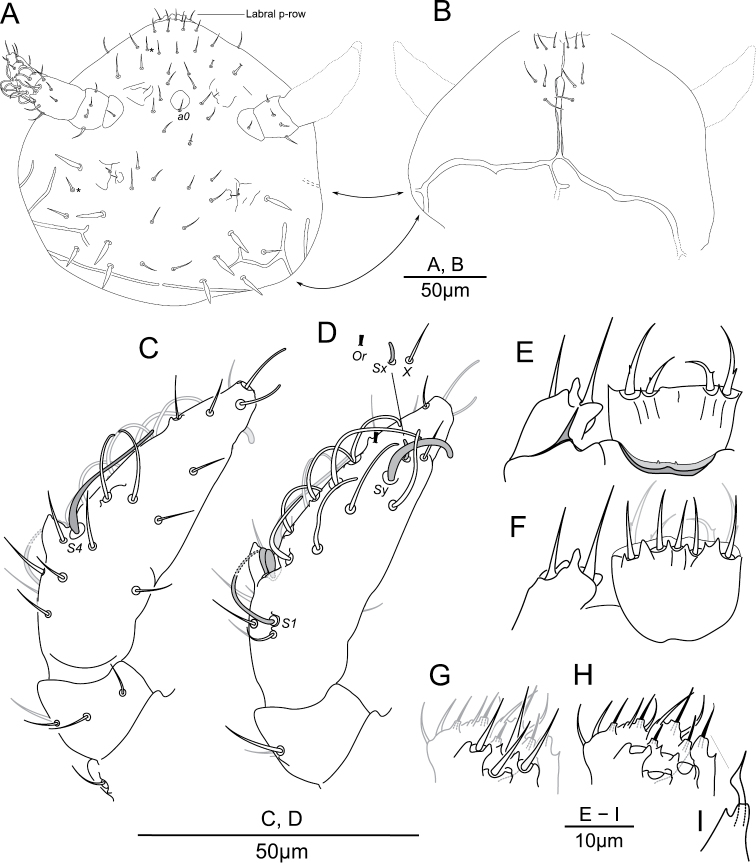
*Megalothorax potapovi* sp. n. Chaetotaxy of head **A** dorsal side **B** ventral side; chaetotaxy of antenna **C** anterior side **D** posterior side; labrum and maxillary outer lobe **E** anterior side **F** posterior side; palp of labium **G** focused on ventral chaetae **H** focused on distal chaetae **I** hypostomal papillate chaeta. * indicates a supplementary chaeta, absent in other specimens.

**Integument.** Secondary granulation made of the usual dorsal rough granules (e.g. Fig. 2A, B) and of smooth and flat irregular discoid granules near the ventral, post-labial chaetae of head. Integumentary channels extending laterally and dorsally in posterior part of head. Those channels as a pair of trees with five terminal branches (Figs 1A, B, 3A, B, 14A). Cephalic channels connection with *linea ventralis* circular (Figs 3B, 14B). Thoracic channels simple, restricted to ventral part.

**Figure 4.**
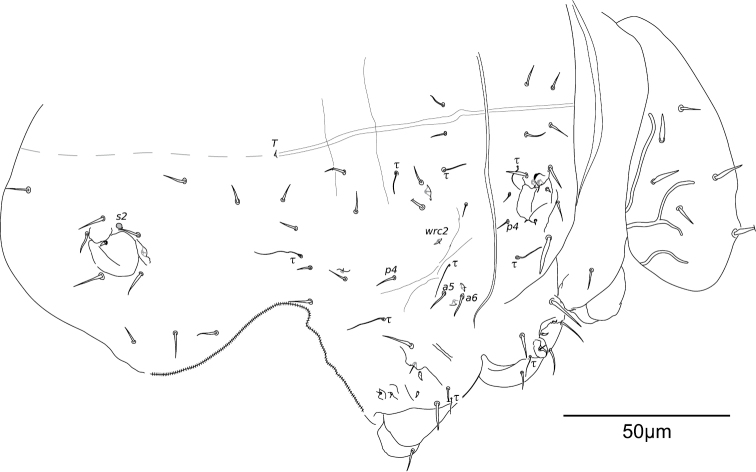
*Megalothorax potapovi* sp. n. Chaetotaxy of trunk, dorsal side.

**Figure 5.**
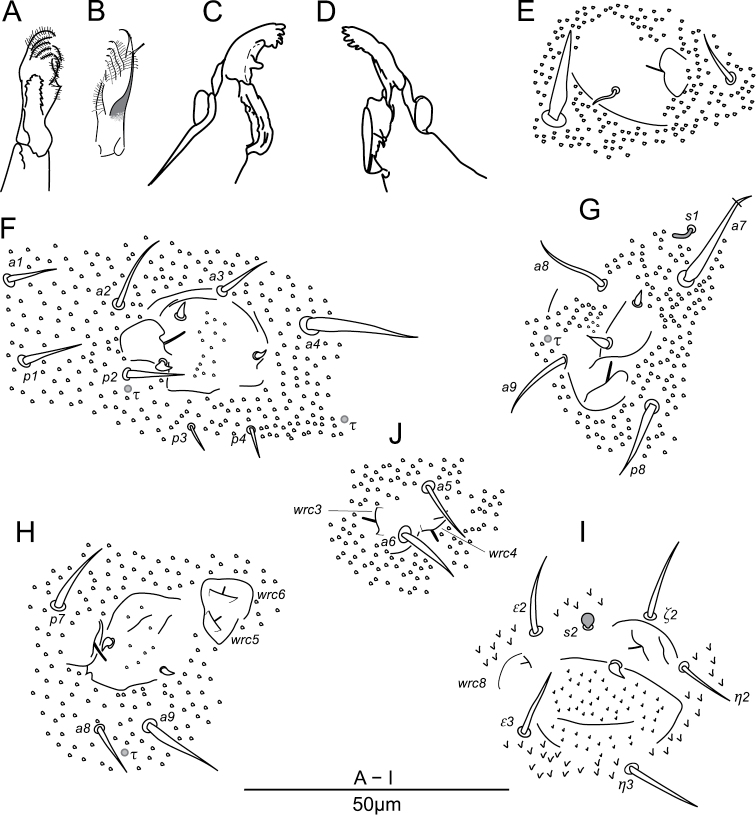
*Megalothorax potapovi* sp. n. Maxilla **A** dorsal side **B** ventral side; mandibula **C** right mandibula **D** left mandibula; sensory fields **E** *sf2* on head **F** *sf3* on Th. II **G** *sf4* on Th. II **H** *sf5* on Th. III **I** *sf6* on abdomen **J** *wrc3*, *4* and chaetae *a5*, *6* on Th. III.

**Sensory fields and wax rods.** A total of 14 + 14 wax rod secretory crypts (2 + 2 on head, 12 + 12 on body), including the ones inserted in each 6 + 6 sensory fields (Figs 3A, 4, 14A, 16A). *sf1* without inner chaeta (Figs 1B, 3A). *sf2* with one rather slender, curved inner chaeta with blunt apex (Figs 1B, 5E). Each inner chaeta of *sf3*–*6* short, flam-shaped and curved (Figs 2A, B, 5F–I). *sf3* with three inner chaetae (Figs 2A, 5F). *sf4* and *5* each with two inner chaetae (Figs 2B, 5G, H). *sf6* with one inner chaeta, inner chaeta length : *sf6* diameter < 0.5 (Fig. 5I). *wrc5* adjoining *sf5* border (Figs 2B, 5H).

**Labrum.** Chaetae (Figs 1D, 3E, F): *a*- and *m*-row with rather slender mesochaetae, *a2* slightly thicker and longer than *m0–2*; *m0–2* apparently smooth, *a1*, *2* with one external teeth and with inward tip; *m0* almost on the same level than *m1*. Integumentary crests (Figs 1D, 13A–C): *m*-row distinctly separated from *a*-row by the antero-median transversal crest (*amt*); longitudinal crest *ml2* projecting anteriorly, cutting the transversal integumentary crest in two part (*amt0*, *amt2*); *mt0* distinctly concave; absence of transversal crest posterior to chaetae *m2* (*pt2*); asymmetry present on an least one specimen: *ml3* strong and projecting anteriorly on one side, *ml3* feeble and not reaching *amt2* on the other side. Anterior side of the anterior process with 3 + 3 and one axial integumentary bulge (Fig. 3E). Labrum ridge with two small pikes (Fig. 3E).

**Other mouth parts.** Oral fold with 2 + 2 mesochaetae. Maxillary outer lobe: palp with subapical mesochaeta and apical papillate macrochaeta, edge of apical papilla with three strong integumentary lobes (Figs 3E, F); sublobal plate with one strong hair (7 μm; Fig. 3E). Basomedian fields of labium with 3 + 3 mesochaetae (Fig. 3B), basolateral fields with 1 + 1 mesochaetae on tubercle. Labial palp chaetal equipment typical of the genus, guard hairs strong in regard of papillate chaetae, hypostomal papillate chaeta flattened laterally in apical part, with subapical enlargement and acuminate apex (Figs 3G–I). Maxilla as in Figs 5A, B. Mandibula each with five apical teeth, right mandibula with a strong tooth between apex and molar plate (Figs 5C, D).

**Head chaetotaxy.** Trend for posterior chaetae to be longer and stronger than anterior chaetae, with 5 + 5 remarkable posterior lanceolate macrochaetae (up to 18 μm, Figs 1B, 3A). Dorsal anterior area with 11 pairs of chaetae (10 + 10 mesochaetae, 1 + 1 macrochaetae) and two axial mesochaetae (Figs 3A, 14A); with an axial integumentary protuberance in front of chaeta *a0*, devoid of secondary granules (Figs 1B, C, 3A); with 2 + 2 indistinct pseudopore-like elements between *sf1* and insertion of antenna. Lateral anterior area with 1 + 1 mesochaetae (Figs 3A, 14A). Dorsal posterior area with 11 pairs of chaetae (5 + 5 lanceolate macrochaetae, 6 + 6 thickened mesochaetae, Fig. 3A, 14A). Ventral side with three pairs of post-labial mesochaetae (Fig. 3B, 14B).

**Antennal chaetotaxy.** Illustrated in Figs 3C, D, pattern diagram in Fig. 15 and summarized in Table 1. Ant. I with one mesochaeta. Ant. II with four chaetae: an anterior mesochaetae and three microchaetae. Ant. III with eight chaetae (four mesochaetae, five microchaetae), two long S-chaetae (*S1*, *S4*) and two short S-chaetae (*S2*, *S3*) in a cupule. *S2* and *S3* protruding from a shallow cupule but partially covered by a strong integumentary lobe. *S1*, *S4* ornamentation unclear in light microscopy, *S2*, *S3* ornamentation feebly visible. *S4* in apical position to *S1*, on the same level than *S2*, *S3*. Tip of *S1* rising up to Ant. IV basal whorl of S-chaetae, tip of *S4* rising up to Ant. IV apical whorl of S-chaetae. Ant. IV with twelve S-chaetae (10 *S*, *Sy* and *Sx*), six microchaetae (only three chaetae in subapical group including chaeta *X*), a small organite (*Or*), two apical and subapical rods (*a*, *sa*). S-chaetae *S* with blunt apex.

**Table 1.** Summary of antennal chaetotaxy.

Ant.	I	II	III		IV			
	chaetae	chaetae	chaetae	S-chaetae	chaetae	S-chaetae	Organit	Sensory rods
*Megalothorax potapovi* sp. n.	1	4	8	*S1–S4*	6 (*X* incl.)	12 (10 *S*, *Sx*, *Sy*)	*Or*	2 (*a*, *sa*)
*Megalothorax sanguineus* sp. n.			9		7 (*X* incl.)			

**Thoracic terga chaetotaxy.** Th. II with 12 + 12 chaetae of variable length, 1 + 1 s-chaetae *s1* tubular and curved and 3 + 3 τ-chaetae (Figs 4, 16A). Chaetae including 3 + 3 macrochaetae (length as *a4* > *a7* > *p8*), 7 + 7 mesochaetae (*a1–3*, *p1* and *p2* thickened, *a8* and *a9* slender), 2 + 2 microchaetae (*p3*, *p4*) (Figs 4, 5F, G). Chaeta *p4* postero-lateral to *sf3* (Figs 4, 5F, 16A). Two τ-chaetae in the periphery of *sf3*, one in posterior position and next to *p2*, one in lateral position and 10–11 granules far from *p4* (Figs 4, 5F, 16A). Th. III with 10 + 10 chaetae, 6 + 6 free wax-rod generating crypts (*wrc1–6*) and 5 + 5 τ-chaetae (Figs 4, 16A). Chaetae including 2 + 2 macrochaetae (*a9*, *p7*), 7 + 7 mesochaetae (*a1*, *a5*, *a6*, *a8*, *p2*, *p3*, *p4*) and 1 + 1 microchaetae (*a3*) (Figs 4, 5H, J). Chaeta *p4* moved posteriorly from *wrc2* (Figs 4, 16A). Chaeta *a6* stronger than *a5* (Figs 4, 5J).

**Figure 6.**
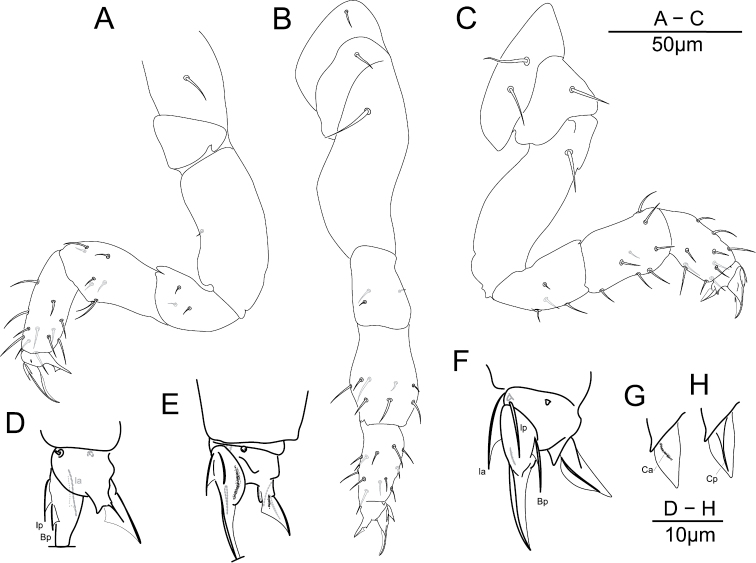
*Megalothorax potapovi* sp. n. Legs chaetotaxy, **A** leg I **B** leg II **C** leg III; claws **D** claw I posterior side **E** claw II anterior side **F** claw III posterior side; unguiculus III **G** anterior side **H** posterior side.

**Legs chaetotaxy.** Legs with ordinary chaetae of variable size as in Fig. 6A–C and summarized in Table 2. Subcoxa 1 I with a mesochaeta, coxa I with a microchaeta. Subcoxa 1, 2 II each with a mesochaeta, coxa II with a macrochaeta. Subcoxa 1, 2 III and coxa III with respectively 2, 1, 1 macrochaetae. Anterior and posterior microchaetae present on each pretarsus.

**Table 2.** Summary of leg chaetotaxy.

Leg	I						II						III					
Segment	Sc1	Sc2	Cx	Tr	Fe	Ti	Sc1	Sc2	Cx	Tr	Fe	Ti	Sc1	Sc2	Cx	Tr	Fe	Ti
*Megalothorax potapovi* sp. n.	1	0	1	3	8	12	1	1	1	3	8	12	2	1	1	4	8	11
*Megalothorax sanguineus* sp. n.																		

**Claws.** Ratio unguis length : pretarsus width on leg I–III respectively as 2.2, 2, 1.73, each claw with ordinary morphology, claw III bulkier than claw I and II (Fig. 6A–C). Each claw subequal in unguis length and in ratio unguiculus : unguis (~0.5) (Fig. 6A–C, F). Unguis basal and posterior auxiliary lamellae (*la*, *lp* and *Bp*) well developed, anterior crest (*Ba*) clear on claw II and III, weaker on claw I (Fig. 6D–F). Each unguiculus with a well developed posterior crest *Cp*, anterior crest *Ca* short and in basal position on claw I and II, more developed and not joining the internal border of the unguiculal lamella on claw III, basal tubercle posterior lobe not or feebly protruding (Figs 6D–H). Ratio unguis length : tibiotarsus length on leg I–III respectively as 0.54, 0.60, 0.65.

**Abd. I–V terga chaetotaxy.** With a total of 18 + 18 chaetae, 1 + 1 τ-chaetae, 2 + 2 free wax-rod generating crypts (*wrc7*, *8*), 1 + 1 globular s-chaetae *s2* (Figs 4, 16A). Chaetae including 17 + 17 mesochaetae (the longest *ε2*, *ε3* and *ζ2* reaching 15 μm) and 1 + 1 macrochaetae (*η3*, 17–18 μm). Chaeta *α3* close to *wrc7*, both clearly anterior to *β3* (Figs 4, 16A). Chaetae *β4* and *ε1* missing.

**Figure 7.**
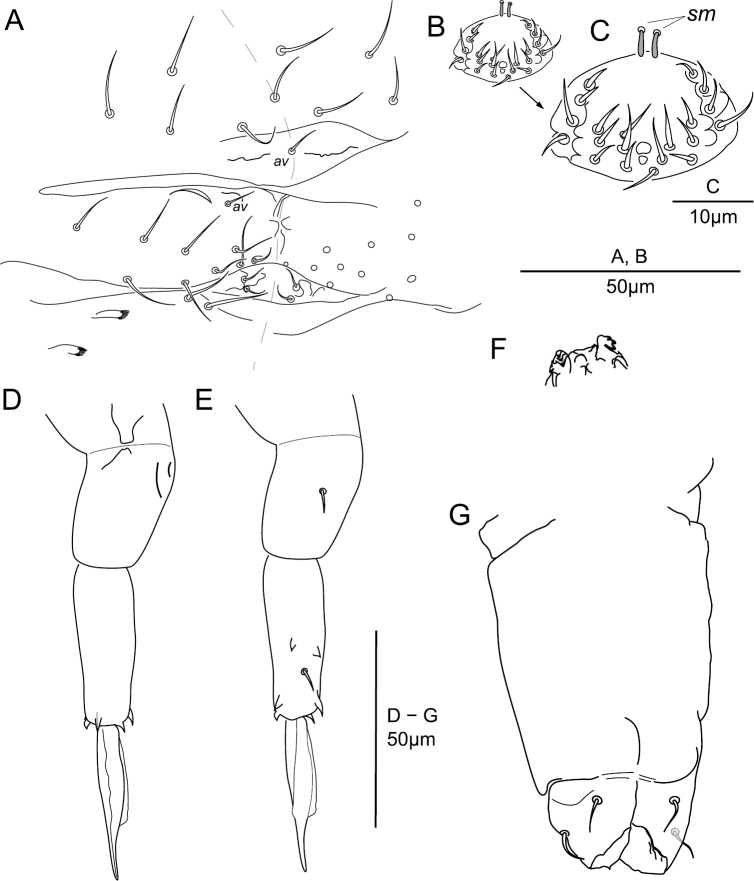
*Megalothorax potapovi* sp. n. **A** Abd. VI and genital plate, female **(B, C)** genital plate, male; furca **D** anterior side **E** posterior side **F** tenaculum **G** ventral tube posterior side.

**Abd. VI and genital chaetotaxy.** Abd. VI: with nine dorsal mesochaetae (Fig. 7A); each anal valve with microchaeta *av* and several granular crests (Fig. 7A); with 9 + 9 ventral chaetae (Fig. 7A), male with 1 + 1 additional ventral cylindrical swollen chaetae *sm* (Fig. 7B, C). Genital plate: female with 2 + 2 microchaetae (Fig. 7A); male with 10 + 10 microchaetae (Fig. 7B, C).

**Abd. IV sternum and furca.** Abd. IV sternum with 2 + 2 neosminthuroid chaetae (Figs 2C, 7A) and 2 + 2 posterior mesochaetae (Fig. 7A). Manubrium with 2 + 2 posterior chaetae. Proximal subsegment of dens with one posterior chaeta (Fig. 7E); distal subsegment posteriorly with two basal spines, one median chaeta and two apical spines, anteriorly with three apical spines, spines without elongated apex (Fig. 7D, E). Mucro with a sharp narrowing in the apical 2/5, lamellae edges smooth (Figs 2D, 7D, E). Ratio dp : dd : mucro = 0.75 : 1 : 0.88; ratio mucro width : mucro length ~0.16.

**Tenaculum and ventral tube.** Tenaculum with 3 + 3 hook-like teeth (Fig. 7F). Ventral tube with two apical pairs of mesochaetae (Fig. 7G).

#### Affinities.

*Megalothorax potapovi* sp. n. has the characteristics of the *minimus* group species (Schneider and D’Haese 2013; Papáč and Kováč 2013). Within this group, it shares with *Megalothorax sanctistephani* Christian, 1998 a median integumentary structure on forehead but differs from it by the presence of the median chaeta *a0*, the presence of the chaeta *X* on Ant. IV, the presence of strong lanceolate macrochaetae on head and thorax and the integumentary channels pattern. The absence of *a5* on Ant. III and of abdominal pair of chaetae *ε1* is a similitude with *Megalothorax svalbardensis* Schneider and D’Haese, 2013 and *Megalothorax tatrensis* Papáč & Kováč, 2013.

The integumentary structure on forehead and the lanceolate macrochaetae clearly separate *Megalothorax potapovi* sp. n. from *Megalothorax minimus*, *Megalothorax aquaticus* Stach, 1951, *Megalothorax svalbardensis*, *Megalothorax willemi* Schneider and D’Haese, 2013, *Megalothorax tuberculatus* Deharveng and Beruete, 1993, *Megalothorax carpaticus* Papáč & Kováč, 2013 and *Megalothorax tatrensis*. Other peculiar characteristics of the species are shape of hypostomal papillate chaeta, presence of a strong lobe protecting *S2*, *S3* on Ant. III, and *S4* in apical position on Ant. III. On the basis of labral features *Megalothorax minimus* (Fig. 13D–F) differs from *Megalothorax potapovi* sp. n. (Fig. 13A–C) by undivided *amt*, *ml2* not joining *amt*, presence of *mt2*. A similar asymmetry was observed in both species (*ml3* joining *amt* on one side, not joining *amt* on the other side). The morphology of the anterior crests (forming the anterior papillae) could not be comprehensively studied.

#### Ecology and distribution.

The species was collected in lowland forest litter, and only found so far in the southern part of Primorye.

#### Etymology.

*Megalothorax potapovi* sp. n. is dedicated to Mikhail Potapov, who led the 2004 field trip in Primorye which allowed to discover the new species.

#### DNA barcode.

A 658bp fragment of the COI gene was amplified and sequenced from paratype (MNHN-EA040229, voucher 00620C05) and seven other specimens (type locality, specimens lost). 251 bases in 5’ were not readable, thus a final sequence of 407bp is available. The sequences are identical. The sequence is deposited into the GenBank database under accession number KR736069. The base composition of the sequence is A = 26%, C = 23.1%, G = 13.5%, T = 37.4% (A + T = 63.4%).

5’– TAAGTTTTTGACTTCTTCCACCTTCTCTCACCCTTCTACTTTCAAGAGGTCTAGCAGAATCAGGTGCTGGAACAGGTTGAACTGTTTATCCTCCTTTATCTTCAAATATTTCCCATGCAGGAGCCTCTGTCGACTTAACTATTTTCAGTTTACATTTAGCTGGTATGTCATCAATTTTAGGAGCTATTAATTTTATTACAACTATCTTTAACATACGATCCCCAGGAATAACATGAGATCAAACTTCACTATTTATTTGATCTGTTTTAATTACATCAATTTTACTTCTCTTGTCTCTTCCAGTTCTAGCAGGAGCTATCACCATACTTTTAACCGACCGAAATTTAAATACTTCATTTTTTGACCCCGCTGGGGGTGGTGACCCAATTTTATACCAACACCTATTC–3’

### Megalothorax sanguineus sp. n.

Taxon classificationAnimaliaCollembolaNeelidae

http://zoobank.org/DD7E3CB4-26AF-4B47-9788-65E2C11F17F4

Figs 8 , 9 , 10 , 11 , 12 , 14C, D , 15 , 16B

#### Material examined.

**Type material.** Holotype: female on slide (MNHN-EA040230), France: Midi-Pyrénées: Ariège: Suc-et-Sentenac: Vicdessos: on the edge of the Bernadouze peat-bog, mosses at a spring to the west under beech; lon=1.4220°E; lat=42.8024°N; alt=1360 m; 13.vi.2013; Berlese extraction, mosses, Lorène Marchal and Anne Bedos leg (09-BDZ1306-G03M) [MNHN]. Paratypes: 2 males and 4 females on slides (MNHN-EA040231–236), same data as the holotype [MNHN].

#### Other material.

4 females on slides (MNHN-EA040237–239]), France: Midi-Pyrénées: Ariège: Saint-Lary: Osque du Couret, forest litter on humid slopes; lon=0.8548; lat=42.8891; alt=1150 m; 28.vii.2010; Berlese extraction, litter, Louis Deharveng and Anne Bedos leg (09-761) [MNHN].

#### Diagnosis.

Reddish in alcohol. Absence of median integumentary protuberance in front of chaeta *a0* on forehead. Presence of chaeta *X* on Ant. IV. Labium: basomedian fields with 3 + 3 chaetae, basolateral fields with 1 + 1 chaetae. Integumentary channels as a paired network on posterior part of the head and a simple channel on anterior part, connection of channels with *linea ventralis* circular. Chaetae on head and trunk with ordinary shape. All inner chaetae of sensory fields 2–6 short flam-shaped. Dorsal abdominal s-chaetae *s2* bean-shaped, absence of dorsal abdominal s-chaetae *s3*. Abd. I to V terga with 20 + 20 ordinary chaetae. Slightly elongated claws. Tenaculum with 3 + 3 teeth. Abd. IV sternum with 2 + 2 chaetae. Mucro lamellae smooth, moderately enlarged.

#### Description.

**General aspect.** Habitus and segmentation typical of the genus. Length from labrum to anus: ~500 μm. Specimens with pale to deep red pigmentation in alcohol. Body chaetotaxy sparse including chaetae, s-chaetae, τ-chaetae as trichobothria, neosminthuroid chaetae, wax rod secretory elements and special swollen chaetae within *sf2*–*6*. Length of chaetae ranging from microchaetae [<6 μm] to mesochaetae [6–10 μm] and macrochaetae [11–15 μm]. Chaetae simple, without any remarkable development.

**Figure 8.**
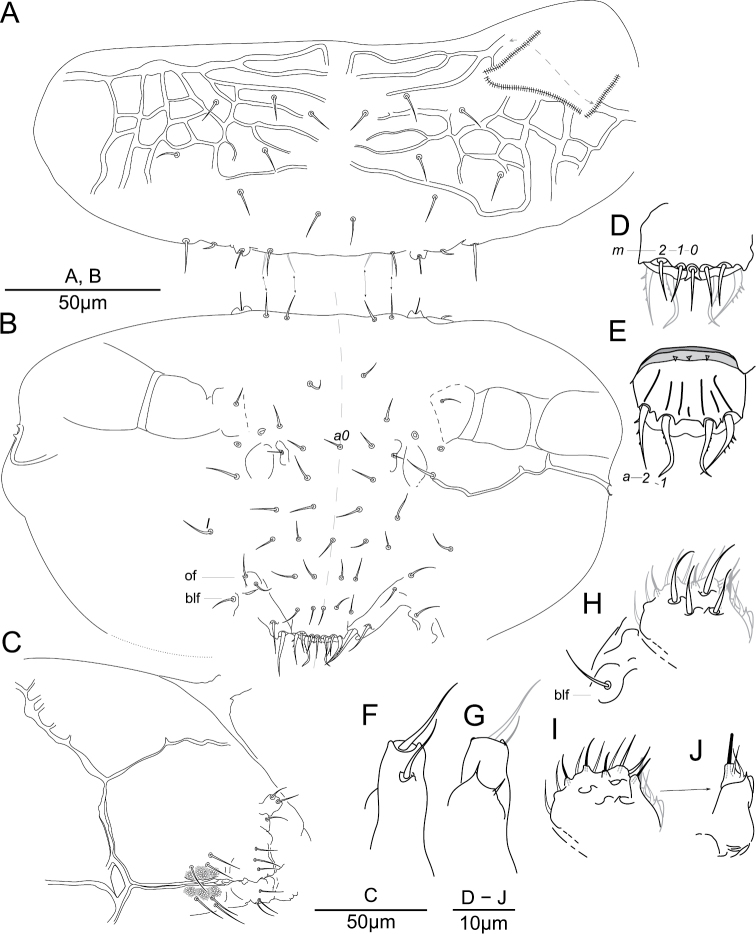
*Megalothorax sanguineus* sp. n. Chaetotaxy of head **A** dorso-posterior side **B** dorso-anterior side **C** ventral side; anterior process of labrum **D** posterior side **E** anterior side; maxillary outer lobe **F** dorsal side **G** ventral side; labium **H** focused on ventral chaetae and basolateral field **I** focused on distal chaetae **J** hypostomal papilla. Legend: blf = basolateral field of labium, of = oral fold.

**Figure 9.**
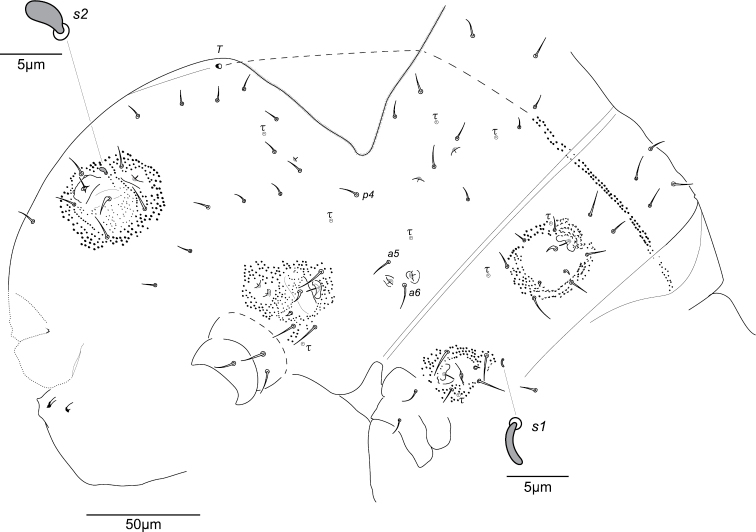
*Megalothorax sanguineus* sp. n. Chaetotaxy of trunk, lateral side. Representation of the granulation limited to the sensory fields area.

**Integument.** Secondary granulation made of the usual dorsal rough granules (Fig. 9) and of smooth and flat irregular discoid granules near the ventral, post-labial chaetae of head (Fig. 8C). Integumentary channels extending laterally and dorsally in anterior and posterior part of head (Fig. 8A, B). Posterior channels as a pair of well developed network. The most detailed observation allowed recognition of at least 10 cycles and 11 terminal branches with unclear tips (Fig. 8A). Anterior channel as a simple branch ending near the lateral edge of *sf1*, touching lateral chaeta of pra.a-row (Figs 8B, 14C). Cephalic channels connection with *linea ventralis* circular (Figs 8C, 14D). Thoracic channels simple, restricted to ventral part.

**Sensory fields and wax rods.** A total of 14 + 14 wax rod secretory crypts (2 + 2 on head, 12 + 12 on body), including the ones inserted in each 6 + 6 sensory fields (Figs 8A, B, 9, 14C, 16B). *sf1* without inner chaeta (Fig. 8B). Each inner chaeta of *sf2*–*6* flam-shaped and curved (Figs 8A, 9), the biggest in *sf6* (Fig. 9). Inner chaeta of *sf6* length : *sf6* diameter < 0.5. *sf2*, *6* with one inner chaeta. *sf3* with three inner chaetae (Fig. 9). *sf4*, *5* each with two inner chaetae (Fig. 9). *wrc5* adjoining *sf5* borders (Fig. 9).

**Labrum.** Chaetae (Fig. 8D, E): *a1*, *2* much thicker and longer than chaetae *m0–2*; *m0–2* smooth, *a2* with three-four external slender teeth and with inward tip, *a1* with three feeble blunt teeth and with outward, flattened tip; *m0* almost on the same level than *m1*. Integumentary crests: *m*-row distinctly separated from *a*-row by the antero-median transversal crest (*amt*); longitudinal crest *ml2* apparently not projecting anteriorly. Anterior side of the anterior process with 3 + 3 clear integumentary bulges and one axial, short bulge (Fig. 8E). Anterior process of the labrum not further studied. Ridge of the labrum with three pikes (Fig. 8E).

**Figure 10.**
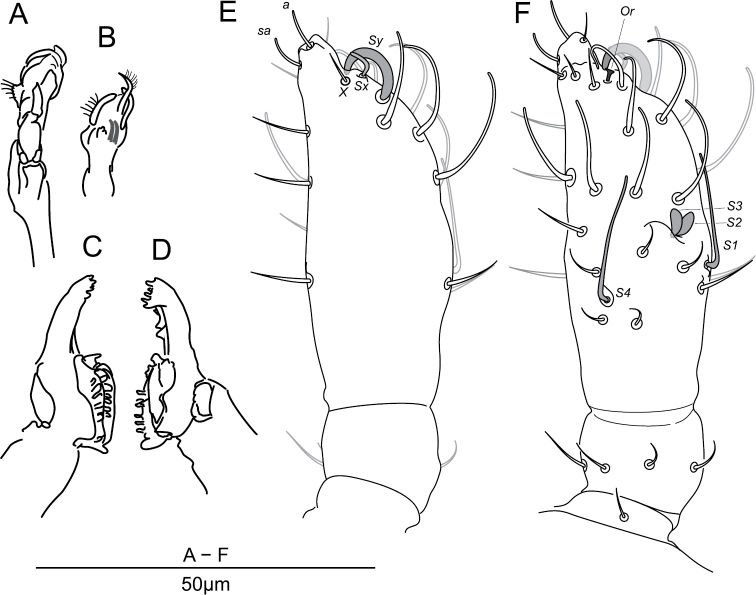
*Megalothorax sanguineus* sp. n. Maxilla **A** dorsal side **B** ventral side; mandibula **C** left mandibula **D** right mandibula; chaetotaxy of antenna **E** ventral side **F** dorsal side.

**Other mouth parts.** Oral fold with 2 + 2 mesochaetae (Fig. 8C). Maxillary outer lobe: palp with subapical mesochaeta and apical papillate macrochaeta (Fig. 8F, G), edge of apical papilla with weak lobes, sublobal plate with two short hairs (Fig. 8F, G). Basomedian fields of labium with 3 + 3 mesochaetae, basolateral fields of labium with 1 + 1 mesochaetae on tubercle (Fig. 8C, H). Labial palp chaetal equipment typical of the genus, guard hairs strong in regard of papillate chaetae (Fig. 8H–J). Maxilla as in Fig. 10A, B. Left mandibula with five apical teeth (Fig. 10C), right mandibula with six apical teeth and a double tooth between apex and molar plate (Fig. 10D).

**Head chaetotaxy.** Dorsally and laterally with mesochaetae, posterior and anterior mesochaetae subequal with a slight trend for posterior chaetae to be stronger than anterior chaetae (Fig. 8A, B). Dorsal anterior area with 11 pairs of chaetae and two axial chaetae (Figs 8B, 14C); with 2 + 2 pseudopore-like elements as ovoid, clear rings between *sf1* and insertion of antenna (Figs 8B, 14C). Lateral anterior area with 1 + 1 chaetae (Figs 8B, 14C). Dorsal posterior area with 11 pairs of chaetae (Figs 8A, 14C). Ventral side with three pairs of post-labial macrochaetae (Figs 8C, 14D).

**Antennal chaetotaxy.** Illustrated in Fig. 10E, F, pattern diagram in Fig. 15 and summarized in Table 1. Ant. I with one mesochaeta. Ant. II with four mesochaetae, anterior chaeta longer than the other. Ant. III with nine mesochaetae, two long S-chaetae (*S1*, *S4*) and two short S-chaetae (*S2*, *S3*) in a cupule. *S2* and *S3* clearly protruding from a shallow cupule, only weakly covered by a feeble integumentary lobe. *S1*–*S4* ornamentation unclear in light microscopy. Tip of *S1* rising slightly above Ant. IV basal whorl of S-chaetae, tip of *S4* rising up to Ant. IV basal whorl of S-chaetae. Ant. IV with twelve S-chaetae (10 *S*, *Sy* and *Sx*), seven ordinary microchaetae, a small organite (*Or*) apically flared, two apical and subapical rods (*a*, *sa*). S-chaetae *S* with blunt apex, rather short (5–6 μm).

**Thoracic terga chaetotaxy.** Th. II with 12 + 12 chaetae of variable length, 1 + 1 s-chaetae *s1* tubular and curved and 3 + 3 τ-chaetae (Figs 9, 16B). Chaetae including 5 + 5 macrochaetae (*a4*, *a7*, *a8*, *p1*, *p8*), 5 + 5 mesochaetae (*a1*, *a2*, *a3*, *a9*, *p2*) and 2 + 2 microchaetae (*p3*, *p4*) (Fig. 9). Chaeta *p4* postero-lateral to *sf3* (Figs 9, 16B). Two τ-chaetae in the periphery of *sf3*, one in posterior position next to *p2*, one in lateral position and 5–6 granules far from *p4* (Figs 9, 16B). Th. III area with 10 + 10 chaetae, 5 + 5 τ-chaetae and 6 + 6 free wax-rod generating crypts (*wrc1–6*; Figs 9, 16B). Chaetae including 4 + 4 macrochaetae (*a6*, *a8*, *a9*, *p7*), 4 + 4 mesochaetae (*a5*, *p2*, *p3*, *p4*) and 2 + 2 microchaetae (*a1*, *a3*) (Fig. 9). Chaeta *p4* moved posteriorly from *wrc2* (Figs 9, 16B). Chaeta *a6* slightly bigger than *a5* (Fig. 9).

**Figure 11.**
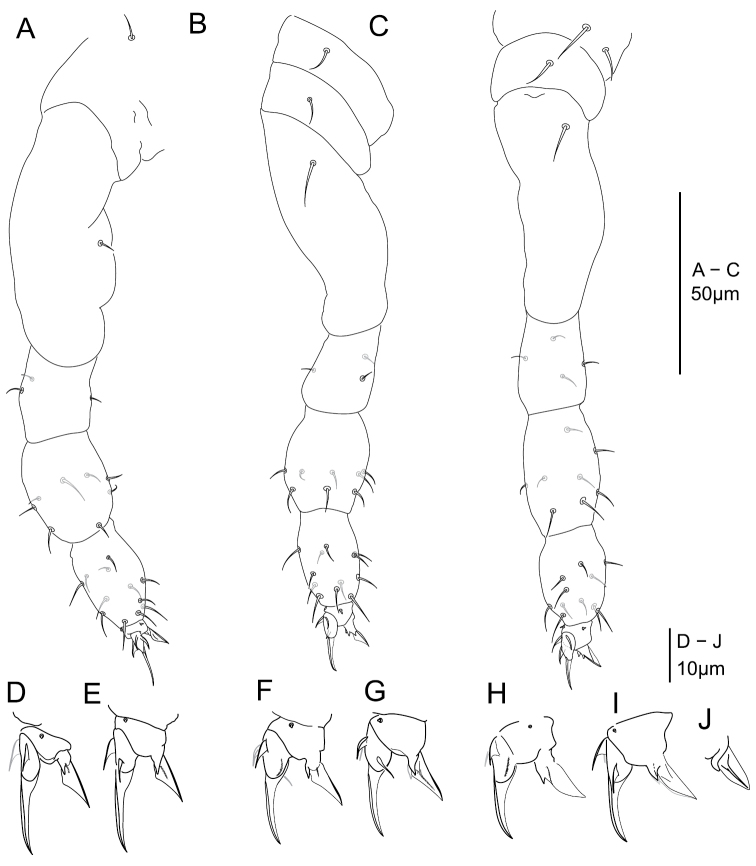
*Megalothorax sanguineus* sp. n. Chaetotaxy of legs **A** leg I **B** leg II **C** leg III; claws **D** claw I anterior side **E** posterior side **F** claw II anterior side **G** posterior side **H** claw III anterior side **I** claw III posterior side **J** unguiculus III interno-posterior side.

**Legs chaetotaxy.** Legs with ordinary chaetae of variable size as in Fig. 11A–C and summarized in Table 2. Subcoxa 1 I with a mesochaeta, coxa I with a microchaeta. Subcoxa 1, 2 II each with a mesochaeta, coxa II with a macrochaeta. Subcoxa 1, 2 III and coxa III with respectively 2, 1, 1 macrochaetae. Anterior and posterior microchaetae present on each pretarsus.

**Claws.** Ratio unguis length : pretarsus width on leg I–III respectively as 3.2, 2, 1.87, claw I with rather slender morphology, claw III bulkier than claw I and II. Claw I with longer unguis and each claw with subequal length of unguiculus, ratio unguiculus : unguis for claw I, II, III as ~ 0.43, 0.5, 0.5 (Fig. 11D–I). Unguis basal and posterior auxiliary lamellae (*la*, *lp* and *Bp*) well developed, anterior crest (*Ba*) clear on claw II and III (Fig. 11F, H), hardly perceptible on claw I. Each unguiculus with a posterior crest *Cp*, anterior crest *Ca* short and in basal position on each claw, joining the internal border of the unguiculal lamella on claw III, basal tubercle with posterior lobe not or weakly protruding (Fig. 11D–J). Ratio unguis length : tibiotarsus length on leg I–III respectively as 1.85, 1.43, 1.47.

**Abd. I–V terga chaetotaxy.** With a total of 20 + 20 chaetae, 1 + 1 τ-chaetae, 2 + 2 free wax-rod generating crypts (*wrc7*, *8*), 1 + 1 s-chaetae *s2* shaped as a bean (Figs 9, 16B). Chaetae including 15 + 15 chaetae rather small and thin (5–7 μm), 5 + 5 stronger chaetae (macrochaetae *ε2*, *ε3* = 11–12 μm, mesochaetae *ζ2*, *η2*, *η3*= 9–10 μm). Chaeta *α3* close to *wrc7*, both clearly anterior to *β3* and *β4* (Figs 9, 16B).

**Figure 12.**
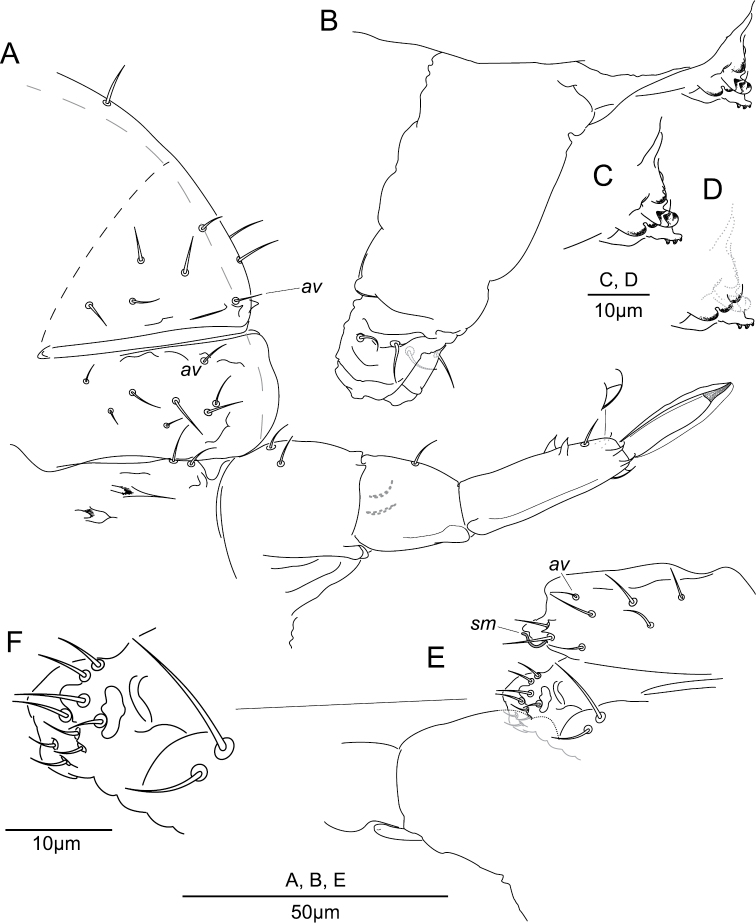
*Megalothorax sanguineus* sp. n. **A** Chaetotaxy of Abd. IV and Abd. VI sterna with furca, female **B** ventral tube and tenaculum, lateral side **C** focus on tenaculum **D** focus on tenaculum axial lobes **E** chaetotaxy of Abd. IV–VI sterna, male **F** focus on genital plate and Abd. IV posterior chaetae, male.

**Abd. VI and genital chaetotaxy.** Abd. VI: with nine dorsal chaetae (6–7 μm) (Fig. 12A); each anal valve with microchaeta *av* and several granular crests (four paired plus one axial on dorsal valve, four on each ventral valve); with 7 + 7 ventral chaetae (4–8 μm; Fig. 12A), male with 1 + 1 additional ventral cylindrical swollen chaetae *sm* (Fig. 12E). Genital plate: female with 2 + 2 microchaetae ; male with 9 + 9 microchaetae (Fig. 12E, F).

**Abd. IV sternum and furca.** Abd. IV sternum with 2 + 2 neosminthuroid chaetae and 2 + 2 posterior mesochaetae (Fig. 12A). Manubrium with 2 + 2 posterior chaetae (Fig. 12A). Proximal subsegment of dens with one posterior chaeta (Fig. 12A); distal subsegment posteriorly with two basal spines, one median chaeta and two apical spines, anteriorly with three apical spines, basal spines without elongated apex, apical spines with elongated apex (longer in posterior spines) (Fig. 12A). Mucro lamellae well developed conferring a slight elliptical shape to the mucro in lateral and dorsal view, with a gradual narrowing in the apical 1/5 (Fig. 12A). Lamellae edges smooth. Ratio dp : dd : mucro = 0.69 : 1 : 75; ratio mucro width : mucro length ~0.23.

**Tenaculum and ventral tube.** Tenaculum with 3 + 3 hook-like teeth (Fig. 12B–D). Ventral tube with two apical pairs of mesochaetae (Fig. 12B).

#### Affinities.

*Megalothorax sanguineus* sp. n. has the characteristics of the *minimus* group species (Schneider and D’Haese 2013; Papáč and Kováč 2013). Within this group, it differs clearly from *Megalothorax sanctistephani* and *Megalothorax potapovi* sp. n. by the absence of a median integumentary structure on forehead. *Megalothorax sanguineus* is similar to *Megalothorax minimus* in terms of chaetotaxic pattern on antenna, legs, and trunk terga (without differences in absence/presence of chaetae). It differs from *Megalothorax minimus* by the shape of the inner chaetae of *sf3*–*7* (some T-shaped in *Megalothorax minimus*, always flam-shaped in *Megalothorax sanguineus* sp. n.), the morphology of chaetae in the dorsal posterior area of head, the integumentary pattern, the morphology of labral chaetae, claw and mucro. The deep red pigmentation of *Megalothorax sanguineus* sp. n. might be similar to that of *Megalothorax rubidus* (Salmon, 1946), but the two species differ in dental spines morphology (the four posterior spines with elongated apex in *Megalothorax rubidus*). *Megalothorax sanguineus* sp. n. shares morphological trends with *Megalothorax aquaticus* and *Megalothorax granulosus* Schneider & D’Haese, 2013: enlargement of mucro lamellae, developed network of integumentary channels on head and elongation of dental spines apex (Stach 1957, Schneider and D’Haese 2013 and pers. obs.). In term of unguis I length : pretarsus I width ratio, it is surpassed by *Megalothorax aquaticus* (epigeic hygrophilous mountains) and *Megalothorax draco* Papáč & Kováč, 2013 (troglobiontic), comparable to *Megalothorax massoudi* Deharveng, 1978 (troglobiontic) and *Megalothorax nigropunctatus* Schneider and D’Haese, 2013 (epigeic, deadwood dwelling); it surpasses slightly *Megalothorax granulosus* (epigeic hygrophilous) and more significantly *Megalothorax tuberculatus*, *Megalothorax hipmani* Papáč and Kováč 2013 and *Megalothorax carpaticus* (troglobiontic). In term of absolute size of the unguis I, it is similar to the two later species, surpasses *Megalothorax granulosus* and is clearly surpassed by *Megalothorax nigropunctatus*, *Megalothorax tuberculatus* and *Megalothorax massoudi* sp. n.

#### Ecology and distribution.

The species is known from humid micro-habitats in Pyrenees, though it was absent from the Bernadouze peat-bog itself. Other *Megalothorax* found in moist mosses in mountains are *Megalothorax aquaticus* (1750m in High Tatras Mountains) (Stach 1957) and *Megalothorax minimus* (up to 1500m in Pyrenees Mountains) (pers. obs.). The combination of morphological features shared with *Megalothorax aquaticus* seems to be related to hygrophilous ecology. In that regard, *Megalothorax sanguineus* sp. n. would remain less morphologically specialized than *Megalothorax aquaticus* but more than *Megalothorax minimus*. “Red” *Megalothorax* are present across the whole Pyrenean range (pers. obs.), and might be *Megalothorax sanguineus* sp. n., but identification has only been confirmed so far for Ariège and Pyrénées-Atlantique specimens.

#### Etymology.

*Megalothorax sanguineus* sp. n. is named after the deep red pigmentation of the species.

#### DNA barcode.

A 658bp fragment of the COI gene was amplified and sequenced from five specimens from the Saint-Lary locality. Specimens were unfortunately lost, sequences identification is based on consistency between: the peculiar pigmentation of the species observed on specimens before destruction, the genetic similarity of the five specimens and the morphological identification of four other specimens with the same pigmentation from the same sample. The sequences are deposited into the GenBank database under accession numbers JN298074–JN298078.

Four sequences are identical (JN298074–JN298077, provided below), base composition is A = 29.6%, C = 17.5%, G = 15.8%, T = 37.1% (A + T = 66.7%). The fifth sequence (JN298078) differs in 11 sites (= 98.3% pairwise identity), base composition is A = 29.5%, C = 17.6%, G = 15.7%, T = 37.2% (A + T = 66.7%).

5’–AACCTTATATTTAATTTTTGGAGTATGATCTGCTATAGTTGGAACAGCATTTAGAGTTTTAATTCGGTTAGAATTAGGACACCCAGGAAGCTTAATTGGAAACGATCAAATCTATAATGTAATAGTTACGGCCCATGCATTTGTAATAATTTTTTTTATAGTAATACCAATAATAATTGGAGGCTTTGGTAATTGATTAGTACCTTTAATAATTGGAGCACCTGATATAGCATTTCCTCGAATAAACAATTTAAGATTCTGACTTTTACCACCATCTTTAATCTTATTACTATCCAGAGGGTTAGTTGAAACTGGTGCTGGCACAGGATGAACAGTATATCCCCCTCTATCGTCTAATATTTCTCATAGAGGAGCTTCTGTAGATTTAACTATTCTTAGACTTCATTTAGCTGGGATATCTTCTATTCTTGGGGCAATTAATTTTATTACAACTATTCTTAATATACGAATACCAGGAATAACATGAGACCAAACTTCTTTATTTGTATGATCAGTTTTTATTACCTCAATTTTATTACTCCTCTCGCTTCCAGTGCTTGCTGGAGCAATTACTATACTTTTAACTGACCGTAACCTGAATACCTCATTTTTTGATCCTGCGGGAGGAGGAGACCCTATTCTATATCAACATTTATTT–3’.

## DNA barcoding results

Fig. 17, Table 3.

**Table 3.** Intra and inter MOTUs genetic distances estimated by Neighbor-Joining with Kimura-2 parameter model.

	**Intra-MOTU distances**																			
Megalothorax cf. interruptus L1	10,70																			
Megalothorax cf. interruptus L2	-	22,98																		
*Megalothorax granulosus*	-	30,02	27,10																	
*Megalothorax minimus*	3,22	38,58	36,01	34,99																
*Megalothorax nigropunctatus*	2,31	28,97	26,91	31,34	29,01															
*Megalothorax perspicillum*	0,00	31,11	30,23	24,91	31,72	26,11														
*Megalothorax potapovi* sp. n.	0,00	29,66	22,89	29,02	27,19	27,91	29,10													
*Megalothorax sanguineus* sp. n.	0,95	29,87	23,49	28,96	33,90	28,19	27,25	25,79												
*Megalothorax* sp1	3,75	35,18	27,46	30,39	25,25	27,74	30,40	23,39	23,36											
*Megalothorax* sp2	-	31,42	26,42	26,02	32,87	29,78	31,61	31,92	32,21	30,97										
*Megalothorax* sp3	-	29,17	27,52	31,81	32,61	31,16	27,84	27,62	27,21	29,64	30,30									
*Megalothorax* sp4	-	28,74	27,23	25,65	27,20	21,90	23,31	24,78	28,56	26,25	23,93	29,61								
*Megalothorax svalbardensis*	-	28,61	26,08	26,72	29,39	26,47	25,67	19,36	22,00	23,83	28,43	29,10	28,38							
*Megalothorax willemi* L1	0,00	27,39	28,66	26,36	27,88	26,69	28,63	22,84	26,89	22,87	32,22	32,80	26,58	23,49						
*Megalothorax willemi* L2	0,00	33,34	32,28	32,22	29,68	32,97	27,64	29,27	29,17	23,80	34,81	33,19	28,69	26,29	21,89					
*Megalothorax willemi* L3	0,52	34,70	35,89	34,40	26,48	31,97	29,36	27,97	34,84	30,04	32,00	37,62	26,26	29,31	24,15	25,08				
*Megalothorax willemi* L4	-	35,46	28,38	37,99	28,84	30,68	32,34	26,23	30,68	24,34	35,00	34,42	27,54	30,71	25,13	24,26	25,45			
*Megalothorax willemi* L5	-	30,24	31,81	32,52	27,27	33,44	29,24	26,12	31,69	26,56	35,48	29,72	30,02	26,05	23,31	26,32	27,08	28,17		
*Megalothorax willemi* L6	0,56	30,64	29,85	28,76	25,65	26,88	26,02	22,39	28,10	21,84	30,24	31,64	27,26	25,06	20,80	25,99	24,20	22,98	20,99	
*Megalothorax willemi* L7	-	32,26	33,03	34,25	26,14	30,37	31,42	23,60	31,72	21,83	31,09	29,61	27,70	26,98	23,08	27,64	26,01	26,68	23,68	20,67

**Table 4.** GenBank accession number.

Specimen	MOTU name	Barcode GenBank accession number
*Megalothorax potapovi* 10770C01 RU120	*Megalothorax potapovi* sp. n.	KR736064
*Megalothorax potapovi* 10770C02 RU120	*Megalothorax potapovi* sp. n.	KR736063
*Megalothorax potapovi* 00620C03 RU120	*Megalothorax potapovi* sp. n.	KR736067
*Megalothorax potapovi* 00620C04 RU120	*Megalothorax potapovi* sp. n.	KR736070
*Megalothorax potapovi* 00620C05 RU120	*Megalothorax potapovi* sp. n.	KR736069
*Megalothorax potapovi* 00620C06 RU120	*Megalothorax potapovi* sp. n.	KR736068
*Megalothorax potapovi* 00620C07 RU120	*Megalothorax potapovi* sp. n.	KR736065
*Megalothorax potapovi* 00620C08 RU120	*Megalothorax potapovi* sp. n.	KR736066
*Megalothorax sanguineus* 6139D02 09761	*Megalothorax sanguineus* sp. n.	JN298074
*Megalothorax sanguineus* 6139D03 09761	*Megalothorax sanguineus* sp. n.	JN298075
*Megalothorax sanguineus* 6139D04 09761	*Megalothorax sanguineus* sp. n.	JN298076
*Megalothorax sanguineus* 6139D05 09761	*Megalothorax sanguineus* sp. n.	JN298077
*Megalothorax sanguineus* 6139D06 09761	*Megalothorax sanguineus* sp. n.	JN298078
Megalothorax cf. interruptus GUF 1	Megalothorax cf. interruptus L1	JN970929
Megalothorax cf. interruptus GUF 2	Megalothorax cf. interruptus L1	JN970928
Megalothorax cf. interruptus GUF 3	Megalothorax cf. interruptus L2	JN970910
*Megalothorax granulosus* cs110_CHL021	*Megalothorax granulosus*	KC900204
*Megalothorax minimus* BEL 1	*Megalothorax minimus*	JN970925
*Megalothorax minimus* cs70_Be001	*Megalothorax minimus*	KC900191
*Megalothorax minimus* cs71_Be001	*Megalothorax minimus*	KC900192
*Megalothorax minimus* cs93_Fr38	*Megalothorax minimus*	KC900195
*Megalothorax nigropunctatus* cd345c	*Megalothorax nigropunctatus*	KC900196
*Megalothorax nigropunctatus* cs104_CHL102	*Megalothorax nigropunctatus*	KC900197
*Megalothorax nigropunctatus* cs118_CHL033	*Megalothorax nigropunctatus*	KC900198
*Megalothorax nigropunctatus* cs119_CHL205	*Megalothorax nigropunctatus*	KC900199
*Megalothorax perspicillum* cs121_Fr114	*Megalothorax perspicillum*	KC900200
*Megalothorax perspicillum* cs122_Fr114	*Megalothorax perspicillum*	KC900201
*Megalothorax perspicillum* cs123_Fr114	*Megalothorax perspicillum*	KC900202
*Megalothorax perspicillum* cs124_Fr114	*Megalothorax perspicillum*	KC900203
*Megalothorax* sp. ARG 1	*Megalothorax* sp2	JN970916
*Megalothorax* sp. ARG 2	*Megalothorax* sp1	JN970926
*Megalothorax* sp. CHL 1	*Megalothorax* sp1	JN970927
*Megalothorax* sp. FRA 8	*Megalothorax* sp4	JN970913
*Megalothorax* sp. USA 1	*Megalothorax* sp3	JN970909
*Megalothorax svalbardensis* cs40_sva19	*Megalothorax svalbardensis*	KC900205
*Megalothorax willemi* ARG 3	*Megalothorax willemi* L6	JN970918
*Megalothorax willemi* ARG 4	*Megalothorax willemi* L6	JN970919
*Megalothorax willemi* cs91_Be005	*Megalothorax willemi* L6	KC900193
*Megalothorax willemi* cs92_Be005	*Megalothorax willemi* L4	KC900194
*Megalothorax willemi* FRA 1	*Megalothorax willemi* L5	JN970912
*Megalothorax willemi* FRA 2	*Megalothorax willemi* L6	JN970917
*Megalothorax willemi* FRA 3	*Megalothorax willemi* L1	JN970911
*Megalothorax willemi* FRA 4	*Megalothorax willemi* L3	JN970920
*Megalothorax willemi* FRA 5	*Megalothorax willemi* L3	JN970921
*Megalothorax willemi* FRA 6	*Megalothorax willemi* L3	JN970922
*Megalothorax willemi* FRA 7	*Megalothorax willemi* L4	JN970915
*Megalothorax willemi* FRA 9	*Megalothorax willemi* L2	JN970924
*Megalothorax willemi* FRA 10	*Megalothorax willemi* L2	JN970923
*Megalothorax willemi* FRA 11	*Megalothorax willemi* L7	JN970914

Twenty MOTUs were delineated using a conservative 19.5% threshold based on the higher bound of the barcode gap (not shown). Over the seven species for which several specimens were sequenced, five were represented by a single MOTU (*Megalothorax nigropunctatus*, *Megalothorax perspicillum* Schneider & D’Haese, 2013, *Megalothorax minimus*, *Megalothorax potapovi* sp. n., *Megalothorax sanguineus* sp. n.) and two exhibited several discrete MOTUs each (*Megalothorax willemi*—7 MOTUs, Megalothorax cf. interruptus—2 MOTUs). Deep genetic divergences showed among the MOTUs (Fig. 17). The mean genetic divergence among the MOTUs was 28.27% (range: 19.36%–37.99%). The mean intra-MOTU divergence was 0.95% (range: 0%–10.70%). The mean observed divergences between *Megalothorax potapovi* sp. n., *Megalothorax sanguineus* sp. n. and the other MOTUs were respectively 26.09% (range: 19.36%–31.92%) and 28.52% (range: 22.00%–34.84%). These ranges of genetic divergences are comparable to those observed among *Megalothorax* MOTUs included in the dataset as well as with the interspecific variation found among MOTUs corresponding to monophyletic identified species (28.08% ; range: 19.36%–36.47%). This supports further the validity of the specific status for the two new species.

## Discussion

### DNA barcoding

The two new species *Megalothorax potapovi* sp. n., *Megalothorax sanguineus* sp. n. are both supported by differences in morphological and molecular characters. The sequencing of COI for a paratype for *Megalothorax potapovi* sp. n. is critical as it will prevent ambiguities if a case of cryptic diversity is discovered in this species (Porco et al. 2012). Indeed, within the genus, the striking example of *Megalothorax willemi* exhibiting a high molecular diversity in parallel with morphological stability calls for a dedicated investigation (Schneider and D’Haese 2013). A similar yet less documented diversity is observed in a new species near *Megalothorax interruptus* (Schneider et al. in prep.).

### Morphology

**Figure 13.**
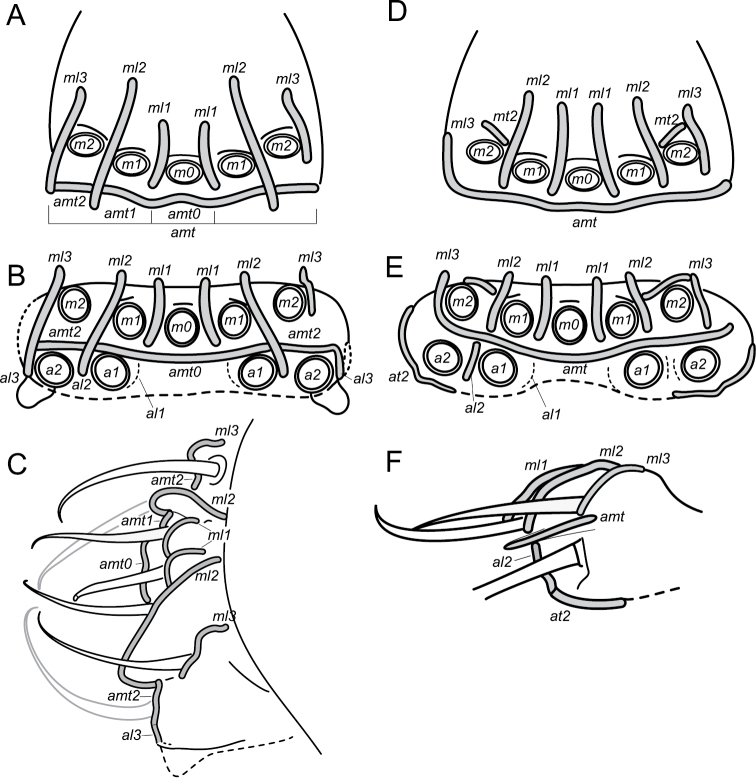
Diagram of the labrum anterior process. *Megalothorax potapovi* sp. n. **A** dorsal view **B** frontal view **C** dorso-lateral view; *Megalothorax minimus* **D** dorsal view **E** frontal view **F** lateral view. In gray integumentary crests, dotted lines indicate areas not clearly observed.

**Labrum.** Schneider and D’Haese (2013) pointed out the potential of the labrum morphology for taxonomy in reporting the differences between *Megalothorax minimus* (*minimus* group) and *Megalothorax nigropunctatus* (*incertus* group). Here some differences are described between two species of the *minimus* group: *Megalothorax potapovi* sp. n. (Fig. 13A–C) and *Megalothorax minimus* (Fig. 13D–F) , and introduce a nomenclature for the integumentary crests of the anterior process of the labrum. This structure remains unpractical to describe comprehensively: in light microscopy, the integumentary crests can be distinguished but their precise development and connections to each other are hard to assess. SEM allowed the description partially and also the recognition of asymmetry, but the method suffers from several flaws: (i) lack of depth, several shots from different angles would be required; (ii) part of the labrum is generally shadowed; (iii) asymmetry being evidenced, several specimens should be observed to assess intra-specific variation. Those requirements prevent use for regular taxonomic due to the cost and availability of SEM equipment, as well as the need for a significant number of specimens.

**Figure 14.**
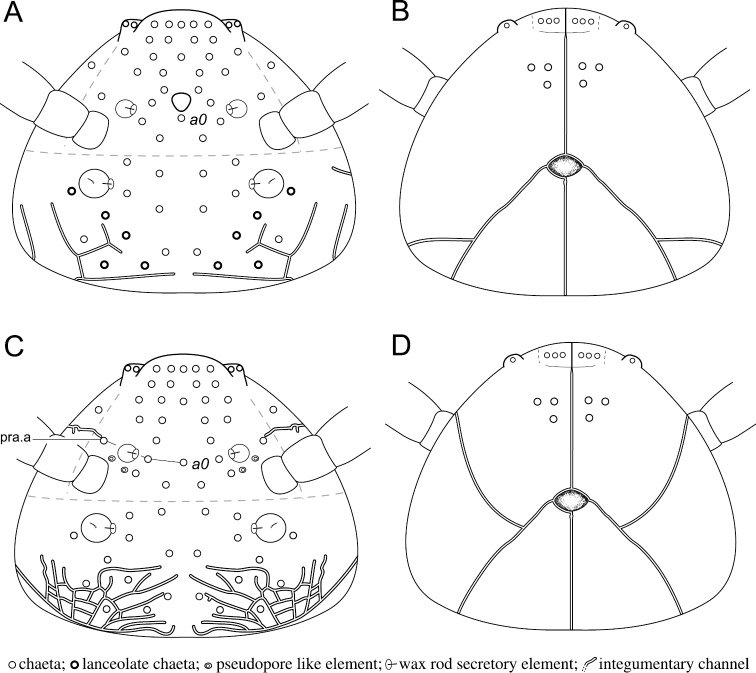
Diagram of the chaetotaxy and integumentary channels network of the head. *Megalothorax potapovi* sp. n. **A** dorsal side (pseudopores not represented) **B** ventral side; *Megalothorax sanguineus* sp. n. **C** dorsal side **D** ventral side.

**Figure 15.**
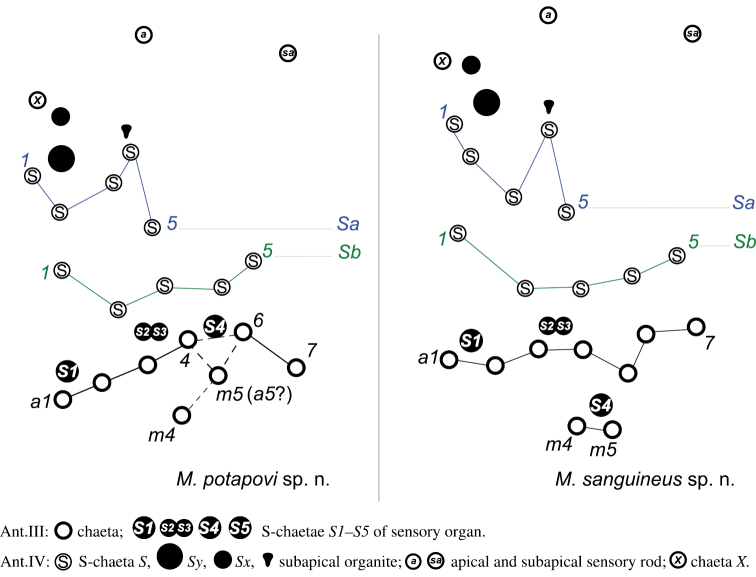
Diagram of antennal III and IV chaetotaxy. *Megalothorax potapovi* sp. n., *Megalothorax sanguineus* sp. n. Alternative hypothesis of homology is indicated in parenthesis.

**Figure 16.**
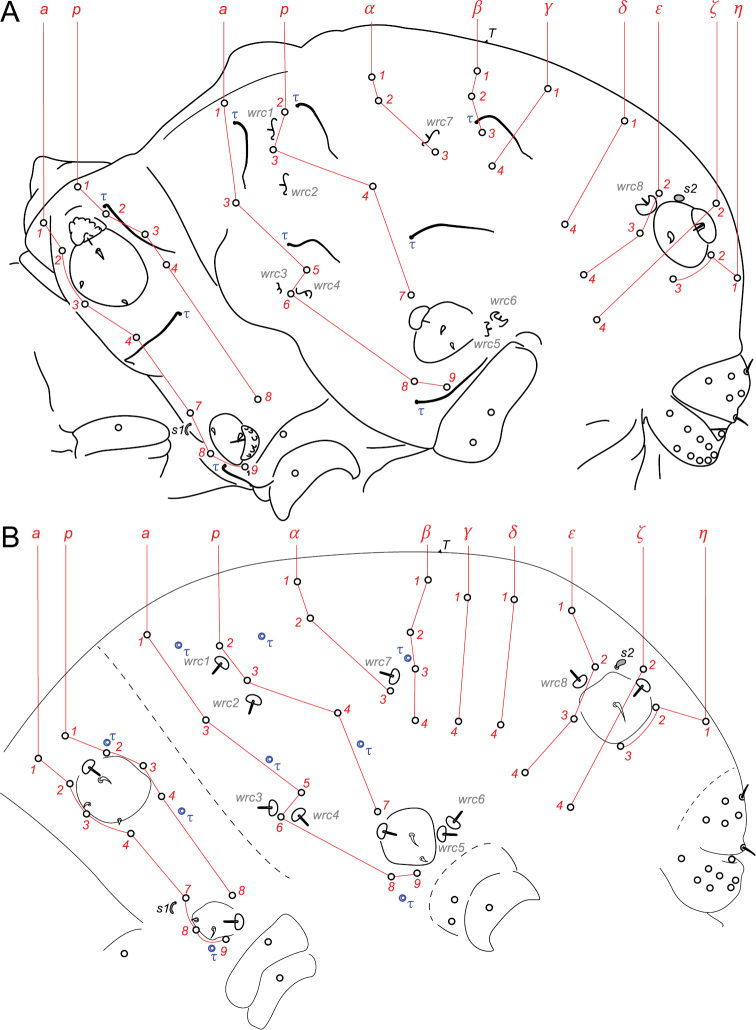
Diagram of the chaetotaxy of the trunk. **A** *Megalothorax potapovi* sp. n. **B** *Megalothorax sanguineus* sp. n.

**Figure 17.**
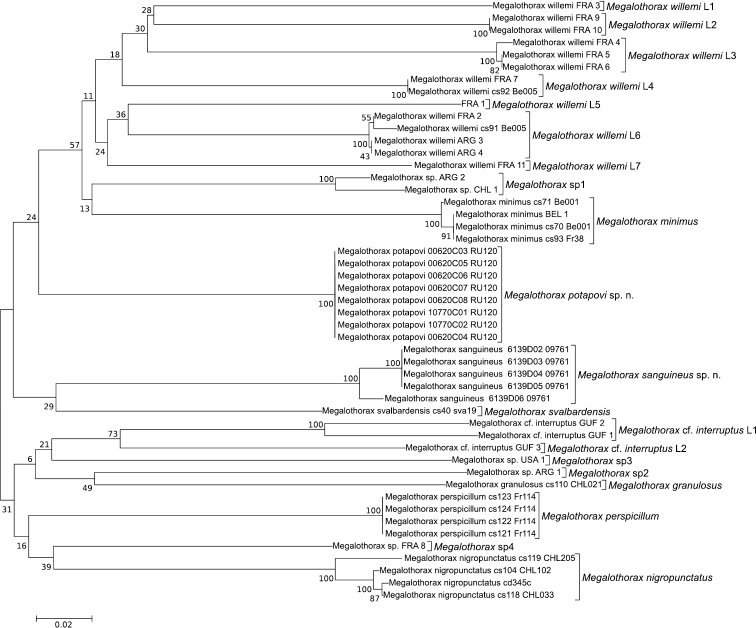
Tree inferred from the COI barcode by Neighbor-Joining with Kimura-2 parameter model. Robustness supports (bootstrap) are reported next to the nodes.

**Integument.** The pairs of pseudopore-like elements at the base of antenna were never reported in the *Megalothorax* genus but seem to be common features of the genus. We observed them clearly on *Megalothorax sanguineus* sp. n. and *Megalothorax carpaticus*, they are visible but faint in the following *Megalothorax* species: *potapovi* sp. n., *minimus*, *granulosus*, *nigropunctatus*, *willemi*, *svalbardensis* and also in French specimens of *Megalothorax sanctistephani*. They were observed in SEM in *Megalothorax perspicillum* and *Megalothorax aquaticus*, where the dorsal one consists of a ring of primary grain and a small integumentary duct and the lateral one of a simple ring of primary grain. At the species level, those characters do not yield evident systematics value. They could not be observed in *Neelus murinu*s and *Neelides folsomi* but the presence of dermastrons could blur the observation of the integument. In the state of knowledge, it is a putative apomorphy of the genus *Megalothorax*.

**τ-chaetae.** Schneider and D’Haese (2013) used the position of the lateral τ-chaetae guarding *sf3* as a descriptor, separating *Megalothorax perspicillum* from *Megalothorax minimus*, *Megalothorax nigropunctatus*, *Megalothorax svalbardensis* and *Megalothorax willemi*. We now describe more precisely the position of the two τ-chaetae guarding *sf3*. Position of the most dorsal τ-chaeta: (i) between *p1* and *p2* and close to *p2* (*Megalothorax minimus*, *Megalothorax svalbardensis*, *Megalothorax willemi*, *Megalothorax tatrensis* and the two new species); (ii) between *p1* and *p2* and equidistant to them (*Megalothorax perspicillum*, *Megalothorax carpaticus*); (iii) between *p1* and *p2* and close to *p1* (*Megalothorax nigropunctatus*); (iv) between *p2* and *p3* and close to *p2* (*Megalothorax granulosus*).

Position of the most lateral τ-chaeta: (i) between *p2* and *p3* (*Megalothorax perspicillum*), (ii) in lateral position and close to *p4* with 2 or less granules between the chaetae (*Megalothorax minimus*, *Megalothorax svalbardensis*, *Megalothorax carpaticus*, *Megalothorax granulosus*), (iii) in lateral position and more or less far from *p4* with 5 or more granules between the chaetae (the two new species and *Megalothorax nigropunctatus*). *Megalothorax draco*, *Megalothorax tatrensis* and *Megalothorax hipmani* were also observed but the τ-chaetae could not be spotted at the exception of the most dorsal τ-chaeta in *Megalothorax tatrensis*.

**Antenna.** The homology of the chaeta directly below *S4* (*m5*) on Ant. III of *Megalothorax potapovi* is ambiguous; an alternative hypothesis is provided in Fig. 15.

**Claws.** The apparent elongation of the claws in *Megalothorax sanguineus* sp. n. called for a comparison with the other species of *Megalothorax*. The ratio of unguis I length : tibiotarsus I width was used by Papáč and Kováč (2013) as an indicator of troglobiontic adaptation. In practice, the tibiotarsus width is not exactly constant along its whole length and is frequently swollen in slide preparations (with dilatation of the integument). We instead estimated the ratio unguis I length : pretarsus I width and the ratio unguis I length : tibiotarsus I length.

## Supplementary Material

XML Treatment for Megalothorax potapovi

XML Treatment for Megalothorax sanguineus
